# Screening approaches and potential of isolated lactic acid bacteria for improving fermentation of *Saccharina latissima*

**DOI:** 10.1186/s12896-024-00926-6

**Published:** 2025-01-05

**Authors:** Evangelia Zioga, Susan Løvstad Holdt, Fredrik Gröndahl, Claus Heiner Bang-Berthelsen

**Affiliations:** 1 Department of Sustainable Development, Environmental Science and Engineering, KTH Royal Institute of Technology, Stockholm, 114 28 Sweden; 2 National Food Institute, Technical University of Denmark, Kgs. Lyngby, 2800 Denmark

**Keywords:** Microtiter plates, Acidification, *Lactiplantibacillus plantarum*, Sugar kelp, Seawed fermentation

## Abstract

**Background:**

With the growing interest in applying fermentation to seaweed biomasses, there is a need for fast and efficient selection of microbial strains that have the ability to 1) acidify quickly, 2) utilize seaweed constituents and c) exhibit some proteolytic activity. The present study aims to provide a fast methodology to screen large bacterial collections for potential applications in optimized seaweed fermentations, as well as investigate and assess the performance of a selected bacterial collection of the National Food Institute Culture Collection (NFICC) in seaweed fermentation. This approach is directed toward high-throughput (HT) methodologies, employing microwell assays for different phenotypical characteristics of lactic acid bacteria isolated from different sources. The overarching aim is the deeper understanding of the selection criteria when designing starter cultures for seaweed fermentation.

**Results:**

By employing high-throughput analytical workflows, the screening processing time is minimized, and among the different strains from a well-characterized strain collection, it was possible to distinguish between strong acidifiers and to replicate similar results when the volumes were scaled from 96-well plates to lab-scale fermentations (40 mL) of whole seaweed. *Lactiplantibacillus plantarum, Lacticaseibacillus paracasei* and, to a lesser extent*, Lacticaseibacillus rhamnosus* were among the fastest strains to reach the lowest endpoint pH values (< 4.5) in less than 48 h. Although the results regarding proteolytic capacity were not sufficient to prove that the candidates can also provide some flavor generation by the cleavage of proteins, NFICC1746 and NFICC2041 exhibited potential in releasing free alanine, glutamate and asparate as free amino acids.

**Conclusions:**

With the described methodology, a large number of terrestrial lactic acid bacteria (LAB) isolates were screened for their performance and possible application for fermentation of brown sewaeeds. With a a fast conversion of sugars to organic acids, three potential new plant-isolated strains from NFICC, specifically *Lactiplantibacillus plantarum ssp. argentoratensis* (NFICC983), *Lacticaseibacillus paracasei* (NFICC1746) and *Lacticaseibacillus rhamnosus* (NFICC2041), were identified as promising candidates for future synthetic consortia aimed at application in bioprocessed seaweed. The combination of such strains will be the future focus to further optimize robust seaweed fermentations.

## Introduction/background

Fermentation has been known for centuries to preserve and enhance organoleptic properties and improve the digestive and nutritional properties of foods [1]. In the context of food, fermentation has emerged as a valuable technological approach for generating a diverse range of novel products using various raw materials, with a particular emphasis on plant-based and marine sources. Among the diverse microbial consortia, lactic acid bacteria (LAB) are present in fermented foods (dairy-, plant- and meat-based) and contribute to the low pH environment through their metabolism of carbohydrates to organic acids [2]. LAB fermentation and subsequent optimizations for foods are well-studied to terrestrial plants and animal-based fermentation, while still underdeveloped within marine sources, specifically macro- and microalgae [3]. Fermenting seaweed might yield promising ingredients for formulating and creating novel foods and nutraceuticals. Unlike most terrestrial plant- and animal-derived products (milk, vegetables, grains, legumes), seaweeds contain challenging fermentation nutrients. Seaweeds are characterized by a high content of bioactive compounds (polysaccharides, polyphenols, and fatty acids), micronutrients and, depending on the species, a good source of protein, among other claims [4, 5]. The challenges that are associated with seaweed fermentation are attributed to the polysaccharide-rich algal cell walls, which are believed to have a hindering effect on their degradation and subsequent biotranformation [6].

Specifically, marine polysaccharides comprise of complex building blocks when compared to other well-known fermentation substrates, such as glucose, galactose, fructose, di- and oligosaccharides [7, 8]. Moreover, brown seaweeds have a relatively low protein content with seasonal variation, for example in the case of *Saccharina latissima* the reported protein content is 3–14% DW [4], and a high content of potentially inhibitory components, such as phenolic compounds, fucoidan among others. Brown seaweed contains a high content of alginates and cellulose [9], which are not digestible by the human digestion tract, thereby considered rich in dietary fibre [10]. As a result of those compositional bottlenecks, lactic acid fermentaion may be difficult to take place with the same success as other substrates.

The utilization of lactic acid bacteria in seaweed fermentation has been focused primarily to the production of biofuels, chemicals, and a limited number of applications for the preservation of freshly harvested seaweed through spontaneous fermentation or the addition of commercial starter cultures [11–14]. However, there is sufficient evidence that seaweed can support the growth of LAB and yeasts with better microbiological stability and improved organoleptic characteristics of the fermented products [15]. Noteworthy findings include potent antioxidant and anti-inflammatory properties associated with the fermentation process [16]. Furthermore, a recent investigation of fermented seaweed rest-raw materials revealed the emerging potential of bacterial metabolism to reducee off-flavors and iodine content, generating this way improved fermentation-based ingredients [17].

In search of suitable bacterial candidates for new substrates, the most common strategy is to isolate new bacterial strains from the substrate of interest. This hypothesis relies on findings that autochthonous microorganisms are already adapted to the substrate composition and are expected to require fewer adaptation experiments [18, 19]. Seaweed-based growth media and fermentation substrates have been reported in the literature, with some examples of employing commercial starter cultures or isolated marine-associated bacteria [11, 20]. However, because dairy-adapted starter cultures often fail to perform at the same rate on substrates with and because of the difficulties in identifying marine LAB, employing strain libraries that were isolated from diverse land environmental niches, such as plants, traditional fermented foods and fecal matter, might be a promising strategy. These adapted lactic acid bacteria are believed to harbor a variety of carbohydrate degradation genes but also exhibit tolerance to common environmental and food-related stresses, for example in the case of brown seaweed salt and antimicrobial compounds (e.g., phlorotannins, sulphated compounds, carotenes, fucoxanthin) [21]. Better starter cultures for food fermentation require well-characterized strains and a better understanding of the genotype/phenotype, with particular interest in the genes involved for desired functions. It is possible to predict phenotypes in a specified context by using genomic data, which makes the screening phase significantly shorter and more reliable [22], however, when screenings are attempted for new substrates, phenotypic characterization is of utmost importance. To facilitate faster and more accurate microbial selection of starter cultures, for single-strain fermentation, several methodologies have been employed. For example, solid agar substrates supplemented with the biomass of interest are usually supplemented with a color indicator to visually confirm the acidification of streaked bacteria [18]. However, such methodologies impose limitations on the sum of simultaneously tested strains when screening a large number of strains is needed. For this reason, miniaturized spectrophotometric 96-well assays offer a rapid analytical workflow that covers the whole array of characterizations that are often useful in bacterial screening.

Building upon the knowledge of former research in seaweed fermentation, the present study aims to contribute to a quick workflow for the selection of potential industrially relevant starter cultures, focused on LAB species. Additionally, the focus is to investigate and assess the performance of a subset of the National Food Institute Culture Collection (NFICC) in seaweed fermentation, which comprises of LAB isolated from various sources. The approach involves high-throughput (HT) methodologies, which employ assay microwell tests for different phenotypical characteristics and performance on a seaweed medium (SM) of the brown seaweed *S. latissima*. The overarching aim is to validate the screening outcome in seaweed suspension setups.

## Materials and methods

### Bacterial strains

**Fig. 1:**
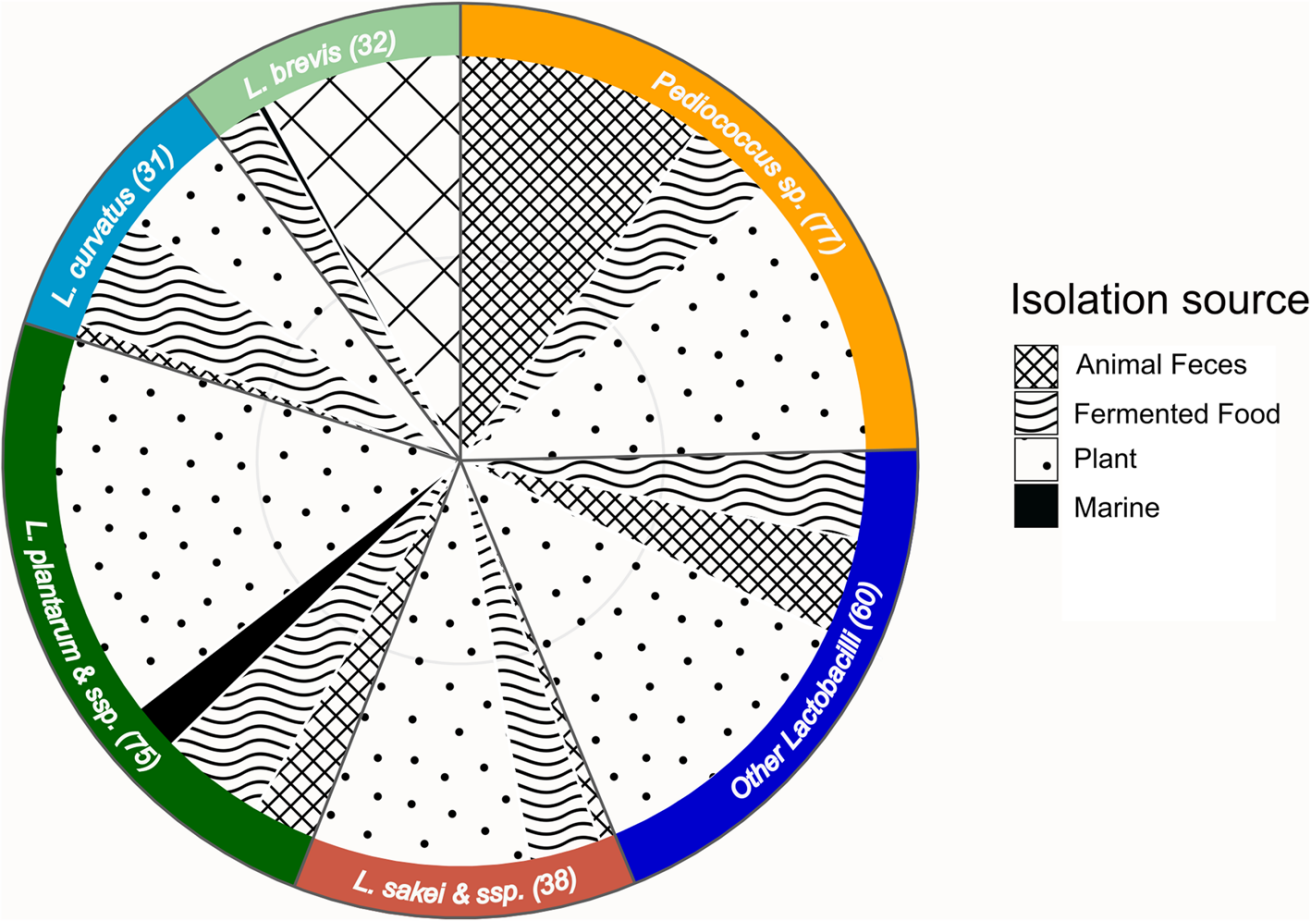
Distribution of strains used in the present study per species and strain. Other *Lactobacillus* species include *L. fermentum*, *L. rhamnosus*, *L. casei*, *L. paracasei*, *L. sanfransiscensis*, *L. delbrueckii*, *L. kimchi*, *L. paralimentarius* and *L. kunkeei*. The isolation source is indicated with the pattern legends

The bacterial strains used in the present study belong to the NFICC from the Technical University of Denmark. NFICC comprises a large selection of bacterial strains isolated from different sources in Denmark, with a special focus on lactic acid bacteria. More than 2000 strains are QPS and belong to *Lactobacillus* sp., *Pediococcus* sp., and *Leuconostoc* sp.*,* among others. A total of 313 strains were screened for their performance as inoculums for seaweed fermentation (Fig. 1). The bacterial isolates were earlier isolated and identified by MALDI Biotyper® Sirius IVD System (Bruker, US) [23]. The strains were revived from the originals in MRS broth (Oxoid LTD, UK) at 30 °C, which were preserved as glycerol stocks (25% v/v) at −80 °C, to create a subcollection on microplates with standardized bacterial concentrations [24]. To create bacterial master plates for the primary screening, the revived strains were streaked twice on MRS agar until single colonies were observed and subsequently incubated in MRS broth. After reaching the exponential growth phase (approximately 18 h), the cells were washed twice in phosphate-buffered saline and centrifuged at 5000 × *g* for 5 min at 4 °C, after which the bacterial concentration was determined by measuring the optical density at 600 nm (OD_600_) in a spectrophotometer (VWR, Radnor, PA, USA). Then, they were diluted to a final OD_600_ of 1 and 15% glycerol, and the master plates were stored at −80 °C until use.

### Seaweed biomass, media and analytical methods

The brown seaweed *S. latissima* was provided in dried flakes from Nordic Seafarm and was harvested from the Swedish Skagerrak coast. Commonly employed biomass characterization analyses were performed, namely, dry matter, ash and total nitrogen with DUMAS combustion (N-protein factor = 5 according to [25]), using a Rapid Max n Exceed (Elementar, Germany). Additionally, total and free amino acids were quantified by LC‒MS as described in Sect. 2.4.1. The soluble fraction of protein in the prepared seaweed media was determined by a Pierce bicinchoninic acid (BCA) assay kit using bovine serum albumin as a standard (Thermo Fisher, Waltham, MA, US).

The medium used for the primary screening was formulated as follows: a seaweed extract was generated by autoclaving a 5% w/v ground seaweed suspension at 121 °C for 15 min. The solids were removed by centrifugation at 8000 × *g* for 15 min at 4 °C, and the supernatant was stored at 4 °C until use. The buffering capacity, monosaccharide content and soluble protein of the seaweed medium (SM) were analyzed. Finally, they were supplemented with 0.5% additional inorganic nitrogen (NH_4_Cl).

The content of monosaccharides, certain disaccharides and primary organic metabolites were determined by UHPLC (Vanquish, Thermo Fisher Scientific, US) with an Aminex HPX-87H column (Bio-Rad, US) coupled with a Shodex RI-101 refractive index detector (Showa Denko K.K., Tokyo, Japan) and a Vanquish Diode Array Detector (Thermo Fischer Scientific, US). The quantification was with external calibration of HPLC-grade standard solutions of glucose, mannitol, fucose, lactic acid, acetic acid, glycerol and ethanol. Chromatograms were processed and analyzed with Chromeleon 6.0 software (Thermo Fisher, USA).

### Screening of lactic acid bacteria phenotypes

#### Acidification rate screening

**Fig. 2:**
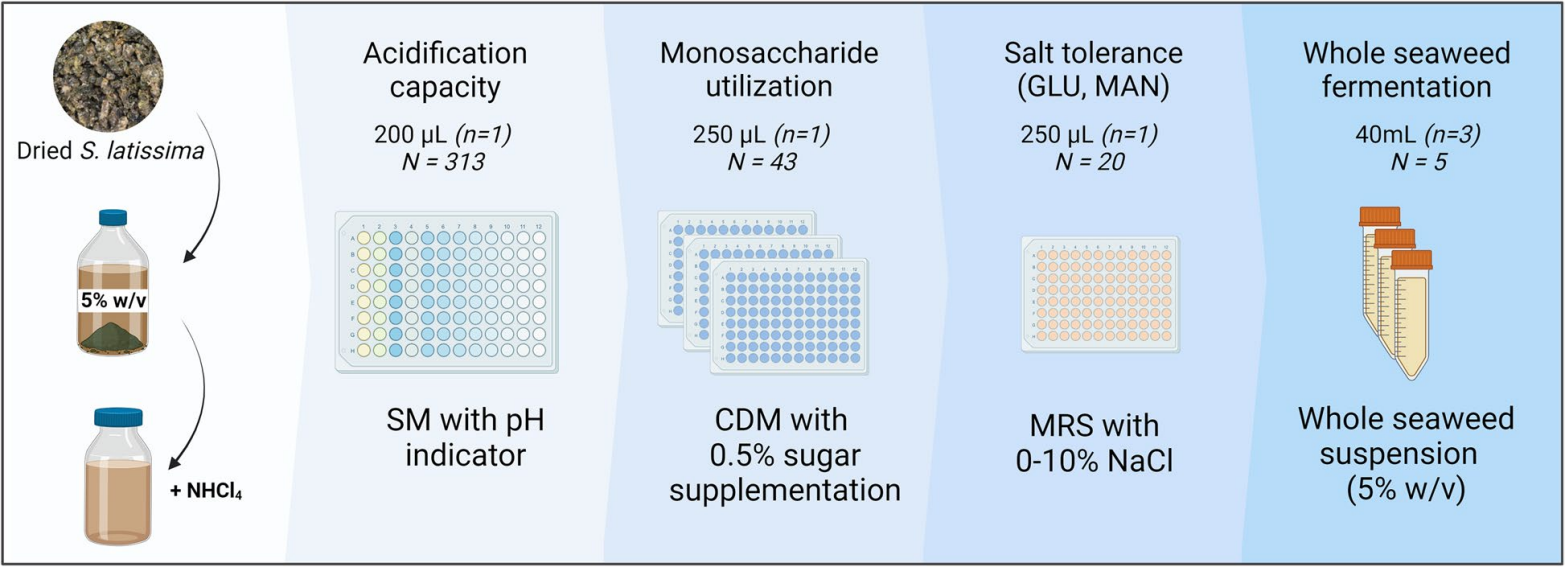
Graphical representation of the screening process. The working volumes, number of replicates (n) and total strains tested (N) are indicated for each screening stage. Figure created with BioRender.com

The selection workflow is depicted in Fig. 2. For the acidification rate screening of single cultures (primary screening), a microwell plate format assay was used with modifications of previously reported methodologies [26, 27]. Prior to the assay implementation, two different color indicators were tested, alone and in mixtures, to determine the best fit of the absorbance signals to pH for the case of SM. Bromocresol Green (BCG) at a final concentration of 0.01 mg/mL was used, and a 12-point pH ladder was created by adjusting the pH of SM_BCG_ with 10% lactic acid. An aliquot of 200 µL was placed in a microwell plate with 1% v/v inoculum. An absorbance scan was recorded from 300–700 nm at 2 nm intervals for each well. Finally, a calibration curve was created at the absorption maximum for BSG (620 nm), and the sum of bacteria in the master plates was incubated at a final OD_600_ of 0.005 in SM_BCG_ at 30 °C for 24 h with constant absorbance monitoring. All the growth experiments were monitored on a Tecan Infinite 200 PRO microplate reader.

#### Sugar utilization profile

Chemically defined media (CDM) devoid of carbon sources were prepared based on the reported compositions from [28] and supplemented with different carbon sources, specifically monosaccharides (glucose, mannitol, xylose, galactose), disaccharides (gentiobiose, cellobiose) and laminarin, as a sole carbon source at 0.5% w/v. Then, the media were aliquoted into 96-well plates and were inoculated with the bacterial master plates at final inoculum concentration of 1% v/v in final volume 250 µL. After 24 h incubation at 30 °C, the OD_600_ was measured and the blank value of the media was subtracted. Glucose values were used to normalize the other sugars, in order to compare the relative growths when other carbon sources are used [24].

#### Salt tolerance

Salt tolerance estimation is essential because seaweeds normally have higher salt content than plant bases. The growth kinetics were calculated after the measurement of growth in standard MRS broth with either glucose or mannitol (0.5% w/v) supplemented with NaCl at concentrations of 2%, 3%, 4%, 6%, 8% and 10% in a 96-well plate for 24 h. The final OD_600_ was 0.01. Media without addition of salt were used as controls, and the absorbance of the media was subtracted from all timepoint values.

### Fermentation and analysis of larger volumes

The selected strains were tested in 40 mL of fermentation media (5% w/v seaweed powder, 2% salt, 0.5% NH_4_Cl). The 50 mL falcon tubes were connected to an iCinac equipment (AMS alliance, Italy), and the pH was monitored for 24 h with 15 min measuring intervals. Samples from three biological replicates were collected aseptically at 12, 24 and 48 h (*n* = 3) and were analyzed for free sugar and organic acid content according to Sect. 2.2 and free amino acids.

#### Free amino acid analysis

For the determination of the free amino acid concentrations, an Agilent 1100 Series LC‒MS apparatus coupled with a bioZen 2.6 μm Glycan column (Phenomenex Inc., USA) and the respective guard column was used. The elution was performed with eluent A (10 mM ammonium formate in acetonitrile) and eluent B (10 mM ammonium formate in water). A mixed solution of the amino acids (Sigma‒Aldrich Production GmbH, Switzerland) was used for the 5-point calibration curve.

Cell- and debris-free supernatants (0.5 mL) were diluted 5 times with buffer (100 mM ammonium formate in water) and subsequently filtered through a 0.22 µm syringe filter (Labsolute, Th. Geyer GmbH & Co. KG, Renningen, Germany) into LC vials with 100 µL inserts. An injection volume of 1 µL was used with a flow rate of 0.5 mL/min for 18 min. The determination was performed in triplicate (*n* = 3). The data analysis of the calibration curves and test samples was performed via Agilent MassHunter Quantitative Analysis software.

## Results and discussion

The species selected for the present study are illustrated in Fig. 1. The subset comprised of different species and isolation sources, as it was intended to include a diversity of metabolic profiles. The main species used were *Pediococcus* sp. and *Lactobacillus* sp., among which 40.6% were categorized as homofermentative, 37% facultative heterofermentative and 22.4% as heterofermentative. Within the *Lactobacillus* spp., the majority of the strains were identified as *Lactiplantibacillus plantarum*, *Latilactobacillus sakei*, *Levilactobacillus brevis* and *Latilactobacillus curvatus*.

The composition of the seaweed biomass used to prepare the SM is shown in Table 1. Sugar kelp, and in general brown seaweed, has a high content of carbohydrates, in the form of storage (laminarin) and structural polysaccharides (alginate, fucoidan, cellulose). In contrast, the protein content in these species is lower than red and green species. According to the BCA assay conducted on the seaweed extract, the soluble protein that was found in the filtered supernatants was as low as 1.012 g/L. It is worth mentioning that there was a low underestimation of protein using spectrophotometric assays on seaweed protein estimations according to earlier observations [29]. The protein content in the seaweed biomass was 8.036 ± 0.045 g/100 g dried seaweed, as calculated by the use of the N-protein factor = 5, which is similar to the total protein from the sum of all amino acids (7.479 ± 0.130 g/100 g dried seaweed). The specific Nitrogen-to-Protein factor for the specific harvest of brown seaweed is 4.65, which can be calculated by the sum of the total amino acids and the total Nitrogen [30].

**Table 1:** Compositional analysis of the dried biomass and seaweed media (SM) of sugar kelp. Each determination was conducted in triplicates (*n* = 3) and is expressed as the mean ± standard deviation (*n* = 3)

Method	Composition
**Dried seaweed flakes**	
DW / Ash (g/100 g)	99.73 ± 0.01 / 97.80 ± 0.14
DUMAS (Protein content) (g/100 g)	8.036 ± 0.045
DUMAS (Total Nitrogen) (g/100 g)	1.607 ± 0,004
LC‒MS (Total protein) (g/100 g)	7.479 ± 0.130
LC‒MS (Free amino acids) (g/100 g)	0.880 ± 0.033
Free carbohydrates (mg/mL)	Mannitol: 7.378 ± 0.304 Glucose: 0.205 ± 0.028 Xylose: 0.07 ± 0.005 Fucose: 0.05 ± 0.002
**Seaweed Media (SM)**	
BCA (Soluble protein) (mg/mL)	1.012 ± 0.003
Free carbohydrates (mg/mL)	Mannitol: 4.904 ± 0.160 Glucose: 0.208 ± 0.005 Fucose: 0.156 ± 0.012

### Evaluation of color indicators and acidification performance

When selecting an optimal starter culture against a new substrate or known substrate with a new type of starter culture, it is important to assess its acidification capacity over time. The traditional use of glass electrodes is indeed a fast and reliable way to determine and monitor the pH in a solution; however, it is not applicable in small volumes usually found in high-throughput methodologies, which are as low as 200 µL. Furthermore, even constant monitoring instruments such as iCinac have limitations in the number of simultaneously monitored samples. An alternative method relies on spectrophotometry (absorbance and fluorescence) with the use of pH-sensitive color indicators [31], which allows the simultaneous monitoring of multiple sammples on plate readers. Precise pH measurements employing spectro-photometry have been under investigation in many fields of natural sciences, such as monitoring of water pH [32, 33] in oceans and plant cultures [34] and enzymatic reactions, and have emerged in fermentation technologies, primarily in high-throughput screening/selection of suitable microorganisms for applications [26, 35, 36]. To the best of our knowledge, such HT methodologies for monitoring pH have not been employed in seaweed fermentation studies. By employing the aforementioned methodology in this study, it was possible to distinguish candidates with satisfactory to remarkable acidification ability among the large number of bacterial candidates.

**Fig. 3:**
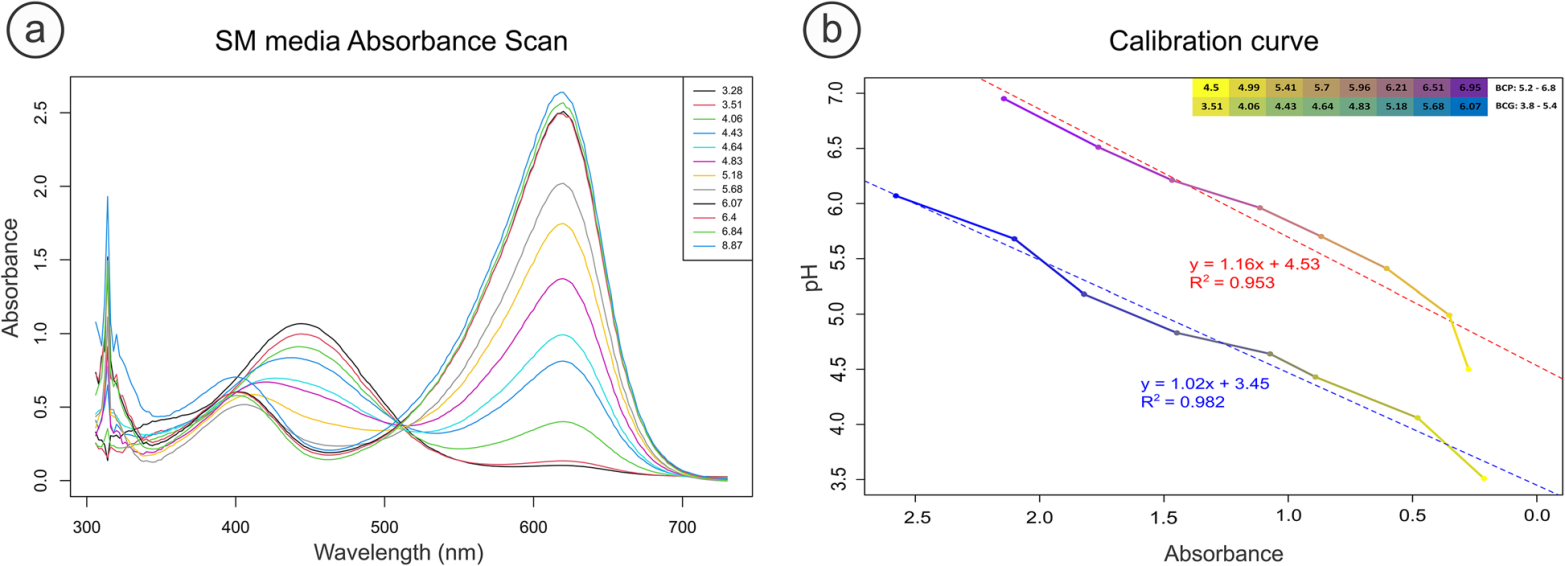
Spectrophotometric data of Bromocresol purple (BCP) and Bromocresol green (BCG) **a**) Absorbance spectra of SM with 0.01% (w/v) BCG at different pH levels. The two wavelength maxima at 440 and 620 nm correspond to the acidic and basic forms of BCG, respectively. **b**) Comparison of the BCP and BCG calibration curves at 0.01% (w/v) in SM and their respecive color change range (top right). The equations and R^2^ are displayed in blue and red for the BCG and BPC, respectively. Each point represents the mean of triplicates ± standard deviation (*n* = 3)

Among the different variations in color indicator mixtures and concentrations, 0.01 mg/mL BCG was found to be the most suitable for this application. The pH color change range of BSG is 3.8–5.4, which is also depicted in the spectral scans of SM_BSG_. Within that range, at 620 nm, the different pH values exhibited characteristic absorbance levels, and no pH points overlapped with each other (Fig. 3a).

By displaying the pH to absorbance, a calibration curve (Fig. 3b) can be constructed to correlate the absorbance at the BCG maximum wavelength (620 nm) and the pH under constant monitoring with a microplate spectrophotometer. The rapid stabilization of pH soon after 5 h in most of the cases (low-growth strains) and the subsequent stabilization of the color in the assay wells, gave a good indication that the assay was robust and that no other factors in the SM interfered with the spectrophotometer readings. In this way, strains could be distinguished between “strong”, “medium” and “weak” acidifying cultures.

The specific criterion to assess the acidification capacity of starter cultures, as proposed based on food safety criteria [2, 14], requires a decrease in pH to less than 4.5, preferably within the timeframe of 24–48 h.

**Fig. 4:**
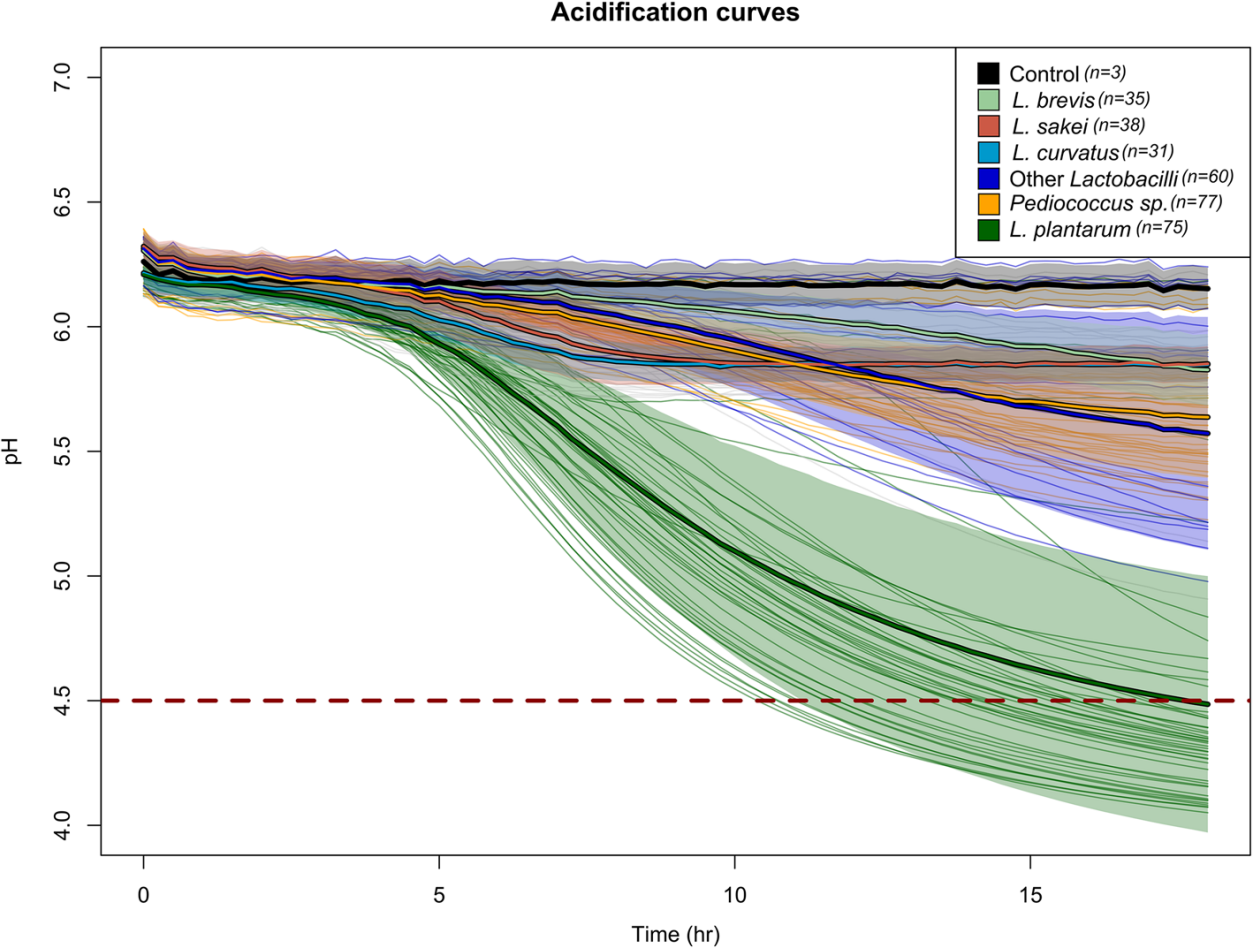
Primary screening of the acidification ability of lactic acid bacteria in seaweed media (SM). Each thin line represents one replicate of the total number of bacteria within species (n) used in the assay (n is mentioned in the legend). The control (uninoculated media) was tested in triplicate (*n* = 3). Bold lines represent the mean of each species group, and the filled colored area represents the standard deviation of all monitored acidification curves per species group

The acidification performance of all strains was monitored in SM with a time cutoff of 18 h (Fig. 4). In this approach, a seaweed extract has been used instead of a synthetic medium, which is formulated by combining carbohydrates and other macronutrients found in brown seaweed, to better mimic the actual food matrix. In this way, components such as polyphenols, micronutrients and cell wall macromolecules are also included in the screening medium, which might give more trustworthy results regarding the ability of bacteria to grow under stressful environments. The primary fermentable sugar alcohol, as indicated by HPLC analysis, was mannitol, and the pH decrease after 18 h was due to lactic acid generation from fermentation due to the consumption of mannitol. In acidification-based experiments, it is possible to confirm the metabolic activity of bacteria. Among the different species, *Lactiplantibacillus plantarum* consistently displayed the fastest acidification, with certain strains reaching the target pH (4.5) in just above 10 h. With some exceptions of strains isolated from fermented foods (kimchi, sourkraut, and sourdough), the majority of fast acidifying strains within these species were of plant origin, primarily root vegetables and animal fecal matter. Plant biomasses have been reported to be a suitable substrate for this species [37], and strains isolated from different environmental niches exhibit remarkable phenotypic diversity. Moreover, bacteria isolated from animals could be a good source of mannitol-utilization bacteria, as it has been demonstrated by some studies [38, 39]. Noteworthy, the five strains isolated from seaweed failed to perform under the experimental conditions with SM at 30 °C. This could indicate that the temperature factor should also be included in the screening procedure, as these strains were isolated at low to ambient temperature (15–20 °C). As Huang et al. [40] and Madsen et al. [18] suggested, most promising starter culture candidates for a specific food substrate should preferably be found naturally in that environment. The next group of strains that showed the ability to grow in the seaweed-based media were strains from the species *Lacticaseibacillus paracasei* and *Lacticaseibacillus rhamnosus*. These strains are mostly associated with dairy environments, however, adapted strains have been isolated from various plants and roots, which was the case for the strains used in the present study. Although the average performance of the sum of all the strains indicated unsuccessful acidification within the target timeframe of 18 h, there were cases of strains that displayed a good acidification rate, such as *L. paracasei* NFICC1746.

*Levilactobacillus brevis* strains showed low acidification capabilities in the SM without any supplementation with additional nutrients. Several recent studies have shown that although *L. brevis* can be isolated from different autochthonous consortia of different seaweed species, evidence shows that it is not the main strain that acidifies the seaweed medium [41]. For the case of *Limosilactobacillus fermentum, Latilactobacillus curvatus* and *Latilactobacillus sakei,* which similarly comprised of various environmental isolation sources, the acidification rates were significantly lower, with an approximate ΔpH of −0.5, which stabilized after 8 h, indicating no further metabolic activity that would generate organic acids and will further decrease the pH.

*L. curvatus* and *L. sakei* are most often related to animal-derived products, especially fermented meat products such as sausages, hams and salami. *L. curvatus* is a facultative heterofermentative microorganism that flourishes in sucrose-rich environments and has shown great potential as an acidifying starter culture but does not play a major role in flavor formation and exhibits exopolysaccharide formation. It also harbors a variety of carbohydrate uptake and degradation genes [42].

Similar trends were reported for *Pediococcus* sp., which included *Pediococcus pentocaseus* and *Pediococcus acidilactici*, that had a significantly slower pH decrease compared to *L. plantarum* and longer lag times (approx. 5 h). From the average slope of the species that follow a slower decreasing rate (*L. brevis*, *Pediococcus* sp.), it is expected that the pH will not drop below 4.5 in the desired timeframe of 48 h. Both species lack the full gene set required for the uptake and conversion of mannitol to the intermediate compound fructose, which then enter the glycolytic pathway.

### Different utilization profiles of selected carbon sources

To obtain better insights into the metabolic capabilities of the strains when grown on single sugars, the growth of 43 selected strains, including both “strong acidifiers” and “low acididifiers” in SM, in a set of selected carbohydrates was determined in minimal media supplemented with 0.5% w/v sugar solution for 24 h. The “strong acidifiers” were mostly *Lactiplantibacillus plantarum* strains when grown in SM, with some exceptions other strains that belong to *Lactobacillus* sp. Regarding the “weak acidifiers”, it was intended to investigate whether the available sugar composition was unfavorable for their growth and which other selected monosaccharides present in seaweed could alternatively sustain growth. Here, it should be noted that the minimal medium composition was based on growth requirements for studies of *Lactobacillus* sp., which might have indicated a different behavior for the case of *Pediococcus* sp. [28].

The most important fermentable sugar was mannitol, which is the main sugar alcohol (alditol) in both its free form and as a monomer in seaweed polysaccharides [9]. The concentration of free mannitol in the SM supernatant was 4.904 ± 0.160 mg/ml, while other mono- and di-saccharides were detected in much lower amounts (Table 1). LAB that are able to utilize mannitol are expected to achieve lower pH values in shorter time, as was demontrated in the earlier acidification curves based on species (Fig. 4). Other sugars tested in the study were glucose, xylose, galactose, cellobiose, gentiobiose and laminarin, since they previously have been found in brown seaweed [11, 14]. The rationale behind the inclusion of the disaccharide cellobiose is that it is the building block of cellulose, which is one of the main structural polymers of algal cell walls. Similarly, gentiobiose, which is a disaccharide with β−1,6 linkage of two glucose molecules was tested to investigate whether the glycosidic bond configurations plays a role in the uptake and to mimic the complex composition of media made of seaweed species [43].

For all screened bacteria, a strain was assessed as growth-positive when it displayed similar growth in CDM supplemented with glucose. However, for some cases, the bacterial growth on glucose was surpassed by that on other carbon sources, for example, in the case of cellobiose, a disaccharide with 2 glucose units, because stock solutions are expressed as g/L of sugar and not glucose equivalents. Very low OD_600_ differences were reported for the case of laminarin-supplemented minimal media; therefore, it was concluded that under minimal media and only laminarin as an available carbon source, the growth of LAB was not sustained. Certain specific enzymes belonging to the family of glycoside hydrolases (GHs) and/or polysaccharide lyases (PLs) are required to first degrade polysaccharides into oligo- and monosaccharides for subsequent utilization by bacteria as carbon sources. The same behaviour was observed for the case of the disaccharde gentiobiose.

**Fig. 5:**
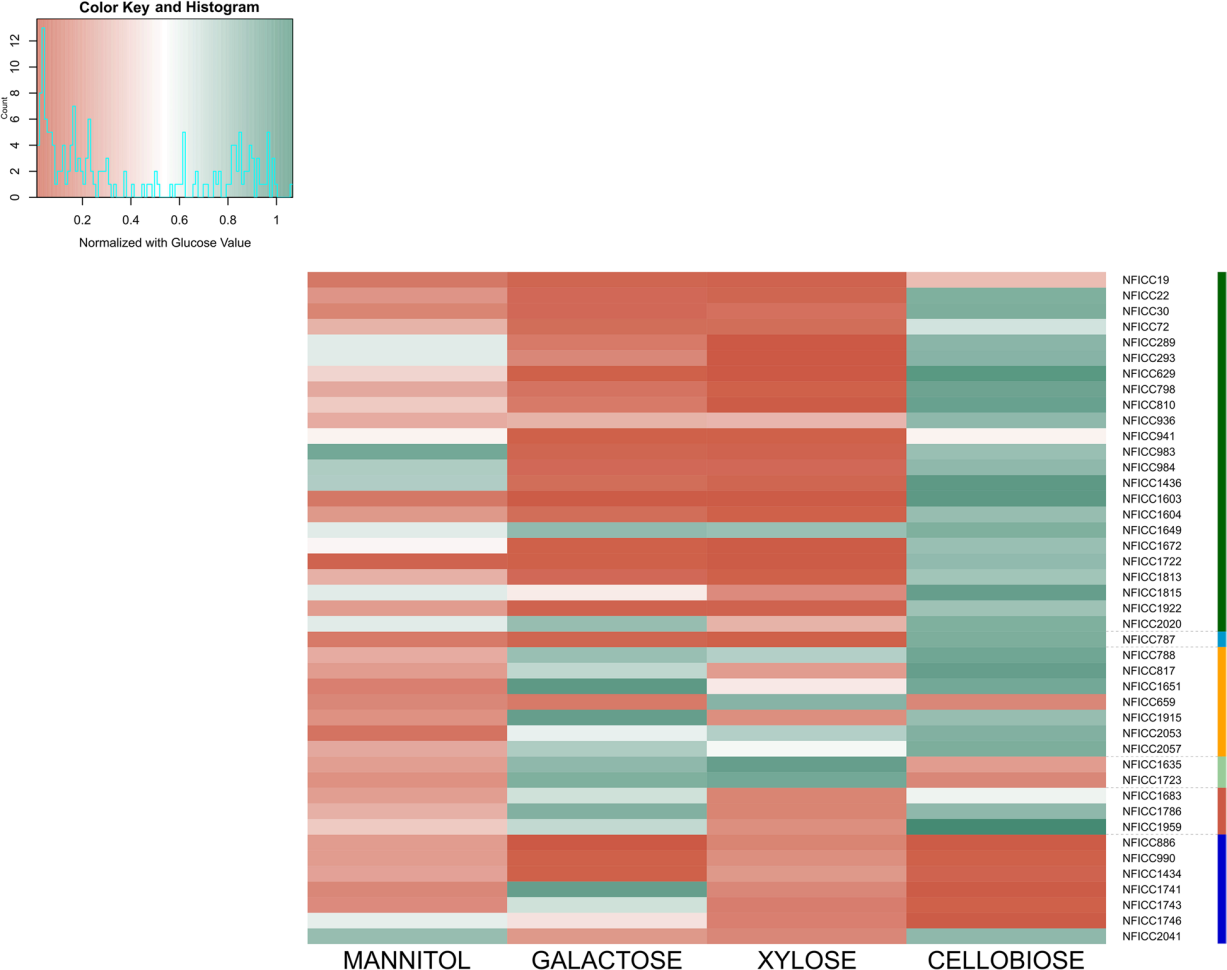
Relative utilization of selected carbon sources (mannitol, galactose, xylose, and cellobiose) in chemically defined media (CDM), normalized with growth on glucose (considered as control) of each of the 43 selected strains. Each column represents one normalized carbon source. Top left: color scale and histogram of strains disctribution. The analysis was conducted with one replicate. The n represents the total number of strains from each species family. *L. plantarum* (*n* = 23), *L. curvatus* (*n* = 1), *P. pentosaceus* (*n* = 7), *L. brevis* (*n* = 2), *L. sakei* (*n* = 3),  Other *lactobacilli *(*n* = 7)

Figure 5 shows the relative growth of the strains tested in the secondary screening normalized by growth on glucose with four monosaccharides as the sole carbon source: mannitol, galactose, xylose and cellobiose. Strains belonging to *Lactiplantibacillus plantarum* (*n* = 23), including subspecies, showed the greatest similarity of the final OD_600_ obtained with mannitol to that obtained with glucose, as did two strains identified as *Lacticaseibacillus paracasei*, namely, NFICC1746 and NFICC2041, accounting for approximately 10% of the total strains tested. Overall, *L. plantarum* strains exhibited good degradation of cellobiose, while most of them lacked the ability to grow on either galactose or xylose. Specifically, regarding mannitol utilization, the relative growth of strains NFICC983, NFICC984 and NFICC1436 were approximately 0.8.

In contrast, strains that exhibited minimal growth on mannitol, specifically *Pediococcus sp*., *L. brevis*, and *L. sakei* and strains NFICC1741 and NFICC1743 (other *Lactobacilli*), exhibited good galactose utilization. Moreover, *L. brevis* strains were among the top 2 strains that grew in the presence of xylose but not in the presence of the disaccharide cellobiose, a behavior that has been reported in studies regarding disaccharide utilization by these species [43]. For *Lacticaseibacillus* sp., the growth pattern on galactose and xylose followed a similar pattern as that of the *L. plantarum* strains, with the exception of NFICC1741 and NFICC1743, which showed good growth on galactose. *L. sakei* strains exhibited good growth on galactose and cellobiose. Strains NFICC886, NFICC990 and NFICC1434 showed insufficient growth compared to glucose in all tested carbon sources. All of them were identified as *Lacticaseibacillus paracasei subsp. paracasei* and were isolated from plant and animal feces. This could be explained either by the incompatibility of the chemically defined media used in the utilization assay or by certain growth parameters that were not met for these strains (e.g., temperature, optimal carbon source).

### Salt tolerance was not affected by the carbon source

Twenty strains were selected based on previous screening experiment to evaluate their halotolerance when they were grown in rich media supplemented with one of two carbon sources and up to 10% salt. The effects of varying salt concentrations combined with mannitol were compared to glucose. In order to develop relevant strategies for biopreservation and further product development in seaweed biomasses, the salt content factor should be considered. Salt is used in spontaneous fermentation and facilitates the suppression of unwanted spoilage microorganisms, while at the same time, added starter cultures that can tolerate broad levels of salt are favored [2]. In the case of seaweed fermentation, the addition of salt can further increase the integrity of seaweed biomass due to osmotic shock when tap water is used, while at the same time allowing better control of fermentation in commercial-scale processes [44].

Table 2 summarizes the growth of the 20 selected strains with glucose and mannitol at four different salt concentrations (0%, 2%, 4%, and 6%). No growth was observed at 8% and 10% salt concentrations*. P. pentosaceus* strains (*n* = 3) exhibited good salt tolerance in both sugars with respect to the lag time. However, the maximum cell density was lower in media that contained mannitol as the sole carbon source. This was not the case for the *Pediococcus acidilactici* (NFICC2057) strain. *Pediococcus* sp. strains have been isolated previously from high-salt environments, which indicates that they are adaptable to stressful conditions, and based on the isolation source, it is possible to obtain strains with better salt tolerance [45]. *Lacticaseibacillus paracasei* strains (*n* = 4) exhibited longer lag times and comparable maximum density levels in both carbon sources, while their growth was suppressed by salt concentrations higher than 2%. All of the strains were isolated from spontaneously fermented Danish vegetables according to [23].

**Table 2:** Lag time and maximum density of 20 different strains in MRS media with 0%, 2%, 4% and 6% w/v NaCl and either glucose or mannitol as the sole carbon source (0.5% w/v for 24 h). The growth on MRS devoid of carbon source was subtracted from the respective OD_600_ absorbance

Species	Strain/Isolation source	C-source (0.5% w/v)	Salt (g/100 mL)	Lag time (h)	Max density (OD_600_)	C-source (0.5% w/v)	Salt (g/100 mL)	Lag time (h)	Max density (OD_600_)
*Pediococcus* sp.	NFICC341 (Brewers’ spent grains)	**Glucose**	0	**4.48**	1.32	**Mannitol**	0	**8.28**	0.73
			2	**5.74**	1.25		2	**8.96**	0.59
			4	**8.10**	1.20		4	**12.91**	0.45
			6	**8.75**	1.08		6	**-**	0.25
	NFICC788 (aminal feces)		0	**5.25**	1.31		0	**4.96**	0.51
			2	**6.00**	1.29		2	**6.30**	0.53
			4	**8.22**	1.25		4	**8.12**	0.41
			6	**11.75**	1.11		6	**12.95**	0.13
	NFICC1651 (sourdough)		0	**4.93**	1.35		0	**4.78**	0.64
			2	**5.44**	1.30		2	**6.47**	0.55
			4	**5.89**	1.28		4	**7.05**	0.51
			6	**11.20**	1.21		6	**12.53**	0.28
	NFICC2057 (flower)		0	**6.00**	1.39		0	**11.50**	0.34
			2	**11.24**	1.35		2	**12.62**	0.29
			4	**15.72**	0.75		4	**-**	0.12
			6	**-**	0.19		6	**-**	0.12
*L. brevis*	NFICC1723 (potato)		0	**5.06**	1.16		0	**-**	0.13
			2	**5.15**	0.88		2	**10.23**	0.33
			4	**6.65**	0.84		4	**15.12**	0.23
			6	**10.10**	0.71		6	**-**	0.18
*L. paracasei*	NFICC1678 (carrot)		0	**8.36**	1.34		0	**8.50**	1.08
			2	**11.95**	1.30		2	**11.02**	0.67
			4	**15.35**	0.93		4	**-**	0.15
			6	**22.97**	0.31		6	**-**	0.13
	NFICC1741 (red bell pepper)		0	**8.35**	1.35		0	**8.48**	0.83
			2	**10.62**	1.19		2	**11.02**	0.73
			4	**12.99**	1.14		4	**-**	0.26
			6	**15.82**	0.84		6	**-**	0.17
	NFICC1743 (red cabbage)		0	**8.13**	1.30		0	**7.20**	0.91
			2	**11.43**	1.17		2	**7.53**	0.83
			4	**12.16**	1.14		4	**-**	0.21
			6	**13.64**	0.89		6	**-**	0.15
	NFICC1746 (pear)		0	**8.37**	1.30		0	**7.96**	0.99
			2	**10.55**	1.19		2	**8.20**	0.84
			4	**13.59**	1.07		4	**-**	0.34
			6	**13.66**	0.84		6	**-**	0.15
*L. rhamnosus*	NFICC2041 (squash)		0	**13.75**	1.31		0	**11.60**	0.76
			2	**15.66**	1.27		2	**12.72**	0.60
			4	**18.98**	0.66		4	**-**	0.17
			6	**-**	0.13		6	**-**	0.12
*L. plantarum*	NFICC22 (sourdough)		0	**3.74**	**1.43**		0	**4.38**	**1.31**
			2	**4.42**	**1.40**		2	**4.91**	**1.27**
			4	**5.31**	**1.25**		4	**5.67**	**1.21**
			6	**8.05**	**1.11**		6	**11.99**	**0.98**
	NFICC30 (sourdough)		0	**3.32**	**1.42**		0	**5.22**	**1.01**
			2	**4.41**	**1.37**		2	**6.87**	**1.21**
			4	**4.96**	**1.25**		4	**9.75**	**1.20**
			6	**7.95**	**1.07**		6	**18.32**	**0.94**
	NFICC72 (gooseberry)		0	**5.76**	**1.42**		0	**5.76**	**1.29**
			2	**5.26**	**1.35**		2	**5.89**	**1.25**
			4	**5.75**	**1.23**		4	**6.15**	**1.23**
			6	**8.52**	**1.09**		6	**13.75**	**0.91**
	NFICC289 (garden plant)		0	**3.66**	**1.50**		0	**4.47**	**1.09**
			2	**3.62**	**1.35**		2	**4.27**	**1.23**
			4	**5.42**	**1.28**		4	**7.51**	**1.20**
			6	**8.50**	**1.15**		6	**15.30**	**1.02**
	NFICC629 (grass)		0	**4.70**	**1.39**		0	**6.02**	**1.29**
			2	**4.71**	**1.36**		2	**5.48**	**1.26**
			4	**6.77**	**1.17**		4	**7.64**	**1.19**
			6	**11.57**	**1.00**		6	**15.38**	**0.82**
	NFICC798 (animal feces)		0	**5.35**	**1.42**		0	**5.17**	**1.31**
			2	**4.42**	**1.36**		2	**6.13**	**1.26**
			4	**5.74**	**1.50**		4	**8.95**	**1.18**
			6	**10.22**	**1.14**		6	**16.26**	**0.88**
	NFICC983 (potato)		0	**4.47**	**1.40**		0	**4.54**	**1.32**
			2	**5.44**	**1.32**		2	**4.84**	**1.25**
			4	**6.52**	**1.22**		4	**5.45**	**1.22**
			6	**9.97**	**1.06**		6	**11.23**	**1.02**
	NFICC984 (potato)		0	**4.54**	**1.43**		0	**5.32**	**0.97**
			2	**6.05**	**1.32**		2	**6.82**	**1.18**
			4	**7.60**	**1.23**		4	**8.80**	**1.22**
			6	**11.75**	**1.07**		6	**17.76**	**0.94**
	NFICC1436 (potato)		0	**5.84**	**1.47**		0	**5.74**	**0.95**
			2	**6.83**	**1.33**		2	**8.59**	**1.20**
			4	**7.83**	**1.22**		4	**11.48**	**1.21**
			6	**12.58**	**1.06**		6	**18.78**	**0.85**
	NFICC1815 (sourdough)		0	**6.75**	**1.36**		0	**4.73**	**1.14**
			2	**7.33**	**1.35**		2	**7.50**	**1.23**
			4	**7.57**	**1.27**		4	**10.31**	**1.09**
			6	**13.35**	**1.09**		6	**19.59**	**0.70**

Although *Levilactobacillus brevis* (*n* = 1) could tolerate salt in glucose-supplemented media, it showed minimal or no growth in the presence of mannitol. *Lacticaseibacillus rhamnosus* (*n* = 1) presented the slowest growth in both glucose and mannitol, while it did not have the ability to exponentially grow at all salt concentrations (Table 2).

*Lactiplantibacillus plantarum* strains (*n* = 10) showed the most potential for entering the growth phase relatively quickly after inoculation with up to 4% salt (3.74–7.64 h) compared to the other strains, with the exception of NFICC1436 and NFICC1815, which were isolated from potato and sourdough, respectively. Interestingly, there was no observed correlation between the isolation source and the growth patterns of these species.

### Fermentation performance in larger scale

Single-strain fermentations of strains that were both characterized as positive-, medium- and low-growth strains were tested in larger volumes of 40 mL of seaweed substrate (5% powder, 0.5% nitrogen, 2% salt) to confirm their performance in autoclaved unfiltered seaweed medium. The pH descrease (ΔpH) and yields of organic acids are depicted in Table 3.

**Table 3:** Identification, isolation source, ΔpH and generation of organic acids of selected strains. The analysis was conducted in triplicate (*n* = 3). The initial pH of the autoclaved seaweed suspension was 6.08 ± 0.13 (*n*= 3)

Strain ID	MALDI ToF Biotyper ID	Isolation	Timepoint	ΔpH	Mannitol (mg/ml)	Lactic acid mg/100 mL	Acetic acid mg/100 mL
NFICC788	*Pediococcus pentosaceus*	Animal feces	24 h	−1.48 ± 0.36	4.51 ± 0.06	28.84 ± 3.43	1.89 ± 0.16
			48 h	−1.96 ± 0.16	3.96 ± 0.11	86.64 ± 4.22	1.96 ± 0.12
NFICC983	*Lactiplantibacillus plantarum ssp. argentoratensis*	Vegetable (Potato)	24 h	−2.13 ± 0.23	3.94 ± 0.11	82.95 ± 0.98	1.33 ± 0.14
			48 h	−2.52 ± 0.17	3.55 ± 0.16	136.51 ± 4.23	1.67 ± 0.22
NFICC1723	*Levilactobacillus brevis*	Vegetable (Potato)	24 h	−1.78 ± 0.21	4.06 ± 0.06	31.24 ± 2.43	22.89 ± 0.92
			48 h	−2.22 ± 0.17	3.66 ± 0.1	99.7 ± 4.85	28.65 ± 0.23
NFICC1746	*Lacticaseibacillus paracasei*	Fruit(pear)	24 h	−1.95 ± 0.13	4 ± 0.07	64.89 ± 3.87	2.18 ± 0.35
			48 h	−2.38 ± 0.26	3.56 ± 0.09	122.95 ± 6.16	2.48 ± 0.16
NFICC2041	*Lacticaseibacillus rhamnosus*	Vegetable(squash)	24 h	−1.82 ± 0.19	4.12 ± 0.11	57.71 ± 3.86	1.29 ± 0.21
			48 h	- 2.16 ± 0.28	3.56 ± 0.1	111.51 ± 8.2	2.65 ± 0.23

Lactic acid was approximately 28 mg/100 mL for the lower acid formation cultures and approximately 83 mg/100 mL for the strong acid formation cultures after 24 h of incubation at 30 °C, that reached a maximum of roughly 136 mg/100 mL in *L. plantarum* NFICC983. As expected, NFICC983 was one of the best performing strains, followed by *Lacticaseibacillus* sp. NFICC1746 and NFICC2041, which were isolated from fruits and vegetables, respectively. The observed ΔpH ranged from −1.48 ± 0.36 to −2.52 ± 0.17, while the pH of the unfermented control did not show significant changes throughout incubation compared to the initial pH (6.08 ± 0.13), as the medium was autoclaved and should not exhibit spontaneous fermentation. Additionally, organic acids were not detected in the unfermented samples by HPLC analysis. An explanation for the ability of *L. plantarum* to thrive in seaweed media may rely on their potential ability to degrade and transform certain polyphenol compounds found in seaweed into derivatives, such as coumaric acid and caffeic acid among others [46]. All strains except NFICC788 descreased the pH below 4.5 within 24 h. The amount of organic acids was further increased by 48 h as shown in Table 3, where all strains exhibited low pH value (< 4.5). As expected, the mannitol content was descreased throughout fermentation, while the small amounts of free glucose (0.208 ± 0.005 mg/ml) were depleted for all strains by 48 h (data not shown in table). Strains NFICC983, NFICC1723, NFICC1746 and NFICC2041 exhibited overall faster mannitol utilization than NFICC788 (19.66% reduction) within 48 h incubation, with 27.6%, 25.37%, 27.4% and 27.4% reduction respectively) compared to the initial mannitol concentration.

Apart from the utilization of carbon sources, and the subsequent generation of organic acids, protein and amino acids metabolism is equally important to determine the performance of food fermentation strains on new substrates. The individual amino acids from the five LAB throughout the fermentation duration are depicted in Table 4. Compared with those in the initial medium (unfermented sample), the free amino acid content in all the samples decreased after 48 h of incubation, while compared to the initial, samplings 12 and 24 showed few statistical differences.

**Table 4:** Amino acid amounts (μg/mL) of initial, 12, 24 and 48 h samplings. All values are represented with the mean ± standard deviation (*n* = 3)

Sample	Timepoint	PHE	LEU	ILE	MET	TYR	PRO	VAL	ALA
**SM**	0	1.08 ± 0.15	4.10 ± 0.61	2.46 ± 0.29	0.68 ± 0.15	5.28 ± 0.37	20.45 ± 0.07	6.08 ± 0.11	162.32 ± 2.13
**788**	12	1.22 ± 0.11	4.43 ± 0.70	2.44 ± 0.32	0.76 ± 0.05	5.20 ± 0.28	20.75 ± 0.26	6.78 ± 0.42 (*)	163.29 ± 3.70
	24	1.25 ± 0.08	3.61 ± 0.66	2.12 ± 0.37	1.24 ± 0.71	4.19 ± 0.34	21.20 ± 0.99	6.36 ± 0.55	172.82 ± 8.86 (*)
	48	0.81 ± 0.09 (*)	7.71 ± 0.15 (****)	1.26 ± 0.14 (****)	0.89 ± 0.06	3.20 ± 0.09 (**)	19.36 ± 0.50 (*)	5.16 ± 0.22 (**)	165.41 ± 1.46
**983**	12	1.12 ± 0.13	4.54 ± 0.75	2.33 ± 0.45	0.48 ± 0.08	5.51 ± 0.23	20.65 ± 0.36	5.92 ± 0.64	163.05 ± 6.07
	24	1.18 ± 0.08	3.56 ± 0.35	1.30 ± 0.13 (****)	0.41 ± 0.08	3.40 ± 0.18 (**)	20.63 ± 0.24	5.54 ± 0.04	169.57 ± 3.61
	48	0.79 ± 0.09 (**)	7.97 ± 0.29 (****)	1.44 ± 0.19 (****)	0.91 ± 0.13	2.94 ± 0.46 (***)	18.98 ± 0.32 (***)	4.13 ± 0.08 (****)	163.23 ± 1.81
**1723**	12	1.30 ± 0.09	4.62 ± 0.21	2.48 ± 0.37	0.60 ± 0.07	4.83 ± 1.02	20.77 ± 0.39	6.06 ± 0.07	163.72 ± 3.56
	24	1.10 ± 0.05	3.43 ± 0.38	1.72 ± 0.71 (**)	0.99 ± 0.19	3.45 ± 0.13 (**)	21.52 ± 0.94 (*)	5.66 ± 0.40	171.82 ± 7.40 (*)
	48	0.87 ± 0.05	8.27 ± 0.72 (****)	1.19 ± 0.17 (****)	1.02 ± 0.16	2.59 ± 0.30 (****)	19.86 ± 0.37	5.06 ± 0.13 (***)	171.01 ± 1.69 (*)
**1746**	12	1.20 ± 0.16	4.27 ± 0.77	2.20 ± 0.28	0.42 ± 0.06	5.02 ± 0.19	20.67 ± 0.24	6.36 ± 0.44	168.62 ± 1.98
	24	1.05 ± 0.07	3.31 ± 0.49	1.55 ± 0.05 (***)	1.28 ± 0.16	3.65 ± 0.44 (*)	21.58 ± 1.14 (*)	5.95 ± 0.46	177.70 ± 9.20 (****)
	48	1.01 ± 0.06	8.74 ± 0.06 (****)	1.33 ± 0.08 (****)	0.82 ± 0.06	4.18 ± 0.36	19.44 ± 0.23 (*)	5.74 ± 0.22	173.72 ± 0.84 (**)
**2041**	12	1.27 ± 0.10	4.46 ± 0.46	2.35 ± 0.27	0.80 ± 0.26	4.84 ± 1.81	20.98 ± 0.32	6.57 ± 0.84	167.92 ± 2.05
	24	1.20 ± 0.22	3.39 ± 0.47	1.81 ± 0.22 (*)	1.12 ± 0.20	3.94 ± 0.18	21.30 ± 0.86	6.35 ± 0.59	176.58 ± 6.99 (***)
	48	1.06 ± 0.08	8.00 ± 0.27 (****)	1.45 ± 0.13 (****)	0.94 ± 0.10	3.98 ± 0.27	19.48 ± 0.36 (*)	6.25 ± 0.21	178.95 ± 4.72 (****)

**Sample**	**THR**	**GLY**	**SER**	**ARG**	**HIS**	**LYS**	**GLU**	**C-C**	**ASP**
**SM**	23.83 ± 1.39	4.63 ± 0.17	5.43 ± 0.28	2.87 ± 0.10	1.25 ± 0.20	12.19 ± 1.01	42.71 ± 0.22	38.77 ± 2.56	65.42 ± 4.22
**788**	25.21 ± 0.93	4.26 ± 0.35	5.27 ± 0.15	n.d.	1.04 ± 0.13	9.25 ± 1.82	42.68 ± 0.91	34.85 ± 5.20	66.32 ± 2.31
	26.66 ± 1.73 (*)	4.36 ± 0.38	5.62 ± 0.38	n.d.	0.91 ± 0.21	2.72 ± 0.10 (****)	43.25 ± 1.72	33.65 ± 3.09	70.29 ± 5.69
	23.39 ± 0.78	4.61 ± 0.20	3.27 ± 0.17 (****)	0.39 ± 0.07 (****)	0.73 ± 0.07	1.96 ± 0.36 (****)	36.76 ± 0.44 (****)	n.d.	52.82 ± 1.22 (****)
**983**	25.25 ± 1.41	3.70 ± 0.14 (***)	4.55 ± 0.64 (***)	2.97 ± 0.03	1.24 ± 0.17	11.50 ± 2.01	40.60 ± 3.44	33.99 ± 5.15	64.92 ± 2.09
	25.60 ± 0.35	3.71 ± 0.14 (***)	2.55 ± 0.13 (****)	2.54 ± 0.12	0.10 ± 0.01 (****)	3.06 ± 0.25 (****)	40.82 ± 1.11	31.88 ± 1.32 (**)	67.36 ± 8.34
	21.70 ± 0.72	4.14 ± 0.16	2.06 ± 0.08 (****)	2.24 ± 0.11	0.57 ± 0.07 (*)	1.14 ± 0.24 (****)	34.90 ± 1.15 (****)	n.d.	49.30 ± 0.46 (****)
**1723**	26.10 ± 1.67	3.71 ± 0.31 (***)	5.03 ± 0.40	n.d.	0.92 ± 0.13	10.08 ± 1.10	44.02 ± 1.19	33.71 ± 1.13	59.92 ± 3.99
	26.48 ± 0.89 (*)	4.13 ± 0.43	3.94 ± 0.38 (****)	n.d.	0.92 ± 0.15	1.28 ± 0.30 (****)	40.11 ± 0.97	31.71 ± 1.31 (**)	64.07 ± 3.88
	22.51 ± 0.51	4.82 ± 0.30	2.79 ± 0.09 (****)	0.30 ± 0.05 (****)	0.59 ± 0.08 (*)	1.52 ± 0.18 (****)	34.47 ± 0.27 (****)	n.d.	52.61 ± 1.52 (****)
**1746**	25.55 ± 0.56	4.23 ± 0.28	5.57 ± 0.15	3.04 ± 0.12	1.00 ± 0.23	7.22 ± 0.83 (***)	46.36 ± 0.72 (**)	36.05 ± 4.19	63.77 ± 2.96
	25.06 ± 2.64	4.42 ± 0.20	5.65 ± 0.36	2.64 ± 0.13	n.d.	n.d.	48.67 ± 2.69 (****)	34.43 ± 4.15	70.65 ± 5.29
	22.51 ± 1.02	4.41 ± 0.17	5.14 ± 0.11	3.79 ± 0.32 (*)	0.65 ± 0.10	n.d.	42.90 ± 0.66	n.d.	59.36 ± 2.03
**2041**	26.00 ± 1.06	4.25 ± 0.48	5.84 ± 0.26	3.35 ± 0.23	1.08 ± 0.14	6.42 ± 1.28 (****)	45.17 ± 0.25	39.10 ± 2.65	66.01 ± 1.71
	25.79 ± 1.25	4.12 ± 0.26	5.35 ± 0.69	3.81 ± 0.24 (*)	0.25 ± 0.03 (***)	1.95 ± 0.37 (****)	48.40 ± 1.73 (****)	35.20 ± 2.20	75.19 ± 1.10 (**)
	23.58 ± 0.55	4.46 ± 0.35	5.24 ± 0.27	3.45 ± 0.14	0.58 ± 0.11 (*)	n.d.	44.26 ± 0.34	n.d.	62.70 ± 0.54

Asterisks represent statistical differences; *p < 0.05, ** *p*< 0.002, *** *p*< 0.0002, **** *p*< 0.0001

The taste-active free amino acids of interest in fermented foods are alanine (ALA), proline (PRO), serine (SER), glycine (GLY), which are chategorized as “sweet” amino acids and the “umami” amino acids, glutamate (GLU) and aspartate (ASP), although dipeptides are believed to exhibit stronger activity [47]. In general, glutamate and aspartate remained at comparable levels after 24 h in the fermentations of NFICC788, NFICC983 and NFICC1723 compared to those in the unfermented sample. However, the free soluble forms of these amino acids were subsequently utilized after 24 h.

The glutamate content of NFICC788, NFICC983 and NFICC1723 showed non-significant changes after 24 h of fermentation, Interestingly, in the case of NFICC1746 and NFICC2041, the free glutamate exhibited a significant increase, at 48.67 ± 2.69 μg/mL and 48.40 ± 1.73 μg/mL respectivelly. Although studied on cheese slurries, findings from Oneca et al. [48] demonstrate that cheese slurries fermented with Lb. paracasei exhibited the highest levels of proteolytic activity, and the umami amino acids GLU/ASP were present in all samples in elevated levels.

Strain NFICC983 diplayed the lowest values of the bitter amino acids histidine and valine after 24 and 48 h respectively, with 0.10 ± 0.01 μg/mL and 4.13 ± 0.08 respectively, as well as phenylalanine (0.79 ± 0.09 μg/mL). Similar decreasing trends have been previously reported earlier in fermented foods and have been associated to the metabolic action of LAB [49]. A similar trend was observed in the case of NFICC2041 were histidine concentrations were reduced by 80% to a final concentration of 0.25 ± 0.03 μg/mL.

Overall, alanine was the most abundant free amino acid in all the samples and decreased the least after 24 h and 48 h, with the exception of NFICC1746 and NFICC2041 that exhibited a significant increasing trend throughout the course of the fermentation. For all tested strains, leucine and isoleucine amino acids displayed opposite trends, where the first increased significantly by 48 h, while the latter decreased. Methionine and Threonine had no significant changed compared to the initial contents.

The role and proteolytic capacities of LAB in nondairy environments are essential for bacterial screening in new fermentation substrates [50]. When moving from the dairy environment to plant bases, nonconventional starter cultures extending from the broadly used *L. bulgaricus* and *S. thermophilus*, which are widely used in fermented milk products, are being investigated. For example, a recent study emphasized the potential of exploring the proteolytic contribution of *L. plantarum* species in food fermentations [51].

Overall, the microtiter-based assays were found to be compatible with seaweed-based media when it is aimed to screen for the performance of LAB in an anaerobic setup. This was possible with the use of a hot water extract of the algal biomass and separation from the solid residue after autoclavation, since the applied heat facilitated the extraction of mainly carbohydrates and other compounds (e.g., proteins, phenolic compounds, minerals). As Allahgholi et al. [9] reported earlier, a hot water treatment was prefered in releasing the water soluble components in the liquid fraction, which could then be used for pH and growth experiments with bacterial inoculums. It is important to mention that the screening media should be devoid of particles that would interfere with the reading in a plate reader, which could lead to false interpetation of results. However, those conditions are unrealistic in actual seaweed fermentations, since the algal biomass would contribute to a dynamic release of compounds during the fermentation duration and thereby influence the microbial metabolism. Therefore, a validation in larger volumes is necessary to confirm the screening results. Indeed, by selecting strains that represent the full range of acidification capacity in the screening phase, it was possible to see similar correlations when grown on seaweed suspensions.

As reported previously, spectrophotometric methods are pivotal in screening experiments, where pH, cell densities and metabolite determination are important criteria for the selection of microorganisms [36]. However, it is important to include more rapid HT assays in the screening phase that could provide information regarding certain phenotypical elements, such as flavor formation (water soluble and volatile compounds), phenolic compound metabolism and antimicrobial action against common pathogenic bacteria.

Here, the screening process, that included acidification capacity in seaweed media, carbohydrate utilization and tolerance to moderate salt concentrations, pointed out five potential candidates to be used in fermentations of brown seaweed. The obtained results from the larger scale fermentations on the actual substrate (seaweed suspensions) showed that while some strains are more active in the faster carbohydrate utilization, and subsequent organic acid production (e.g., 82.95 ± 0.98 mg/100 mL lactic acid in *L. plantarum* NFICC983), other strains hold potential in proteolytic activities and release of free amino acids (e.g., highest concentrations of GLU and ASP in fermentations of NFICC1746 and NFICC2041). Harnessing different phenotypic elements and combining strains (starter and non-starter) into a consortium would facilitate a more robust fermentation performance. Therefore, further research should be directed toward including more selection criteria in the screening process and assessing co-fermentations in both microtiter and larger-scale setups.

## Conclusions

In search of suitable starter cultures that allow the rapid acidification and modification of algal biomasses, there is a need for faster screening methods that are applicable to the biomass of interest. In this study, the aim was to investigate whether, by employing rapid spectro- photometric methods, it is possible to screen large bacterial collections to find optimal strains that could achieve good performance for applications in seaweed acidification. To do this, a pool of 313 LAB strains from the NFICC was screened, and as a first step, the acidification performance was determined within 18 h using a pH color indicator. The use of the color indicator BCG was suitable for the clarified seaweed extract that was used in the high-throughput methodologies. Similarly, the abilities of the strains to tolerate salt and utilize monosaccharides that are found in seaweed were measured in microtiter assays. Consistent with previous studies, the most potent bacteria with regard to acidifying ability were *Lactiplantibacillus plantarum* isolates. However, the strains showed diverse growth preferences when grown in SM and in single sugars, which indicates that it is not a prerequisite that strains from these species will perform the same in seaweed suspensions. Overall, the different species exhibited various utilization patterns when grown in sugars that can naturally be found in seaweed substrates. Five strains were tested in larger volumes and exhibited similar acidification performance in the presence of algal biomass compared to SM. Free amino acid determination revelaed the potential of species within *Lacticaseibacillus* genus to exhibit proteolytic activities when grown on seaweed substrates. Alanine, glutamate and aspartate were the most abundant free amino acids in the seaweed suspensions, which when fermented with NFICC1746 and NFICC2041 their contents reached the highest concentrations compared to the unfermented at 177.70 ± 9.2 /178.95 ± 4.72 μg/mL, 48.47 ± 2.69/ 48.40 ± 1.73 μg/mL and 74.19 ± 1.10 μg/mL (NFICC2041 only) respectively. Further research should be directed toward combining different phenotypes into a synthetic starter culture to ensure resilience in unfavorable substrates such as seaweed species.

## Data Availability

The datasets generated and analyzed during the current study are available from the corresponding author on reasonable request.
